# Joint Exposure to Chemical and Nonchemical Neurodevelopmental Stressors in U.S. Women of Reproductive Age in NHANES

**DOI:** 10.3390/ijerph110404384

**Published:** 2014-04-22

**Authors:** Amanda M. Evans, Glenn E. Rice, Linda K. Teuschler, J. Michael Wright

**Affiliations:** 1 Oak Ridge Institute for Science and Education (ORISE), U.S. Environmental Protection Agency (EPA), Office of Research and Development, National Center for Environmental Assessment, Cincinnati, OH 45268, USA; 2 U.S. EPA, Office of Research and Development, National Center for Environmental Assessment, Cincinnati, OH 45268, USA; E-Mails: Rice.Glenn@epa.gov (G.R.); Wright.Michael@epa.gov (M.W.); 3 Linda Teuschler and Associates, St. Petersburg, FL 33707, USA; E-Mail: lindateuschler@gmail.com

**Keywords:** allostatic load (AL), chronic stress, cumulative exposure assessment, cumulative risk assessment (CRA), hazard index (HI), nonchemical stressors, neurodevelopment, physiological dysregulation, susceptibility, vulnerability

## Abstract

Lead (Pb) and methyl mercury (MeHg) are well established neurodevelopmental toxicants (NDTs), but joint exposure to chemical and nonchemical (e.g., maternal stress) stressors has rarely been considered. We characterized exposure to Pb, MeHg and a measure of physiological dysregulation associated with chronic stress and examined race/ethnicity as a predictor of joint NDT exposure. Using data from the 2003−2004 NHANES, potential chronic stress exposure was estimated using allostatic load (AL), a quantitative measure of physiological dysregulation. A Hazard Index was calculated for joint exposure to Pb and MeHg (HI_NDT_). Logistic regression was used to assess the relationship between an indicator of elevated joint NDT exposures (HI_NDT_ > 1) and race/ethnicity. The multivariate model was stratified by AL groups to examine effect measure modification. African American (adjusted odds ratio [OR] [95% confidence interval] = 2.2 [1.4, 3.3]) and Mexican American (1.4 [0.7, 2.6]) women were more likely to have an HI_NDT_ > 1 compared to Caucasian women. Chronic stress was identified as an effect measure modifier with the largest ORs among women with high AL scores (African Americans = 4.3 [2.0, 9.5]; Mexican Americans = 4.2 [1.3, 14.1]). Chronic stress was found to modify the association between elevated joint NDT exposure and race/ethnicity, highlighting the importance of evaluating chemical and nonchemical stressor exposures leading to a common endpoint.

## 1. Introduction

*In utero* exposures to neurodevelopmental stressors, both individually and in combination, are public health concerns [1,2]. Although lead (Pb) and methyl mercury (MeHg) are known human neurodevelopmental toxicants (NDTs) at low exposure levels [3,4], few studies have examined their co-exposure [5] or joint toxicity [6]. Maternal prenatal stress also may adversely affect neurodevelopment [7].

Certain populations may be at increased risk of elevated exposure to NDTs and chronic stress. For example, African Americans have exhibited elevated blood Pb levels [8] and elevated chronic stress exposure [9]. Limited human studies suggest interactions between Pb and stress [10]. Although none of these epidemiological studies focused on reproductive-aged or pregnant women, toxicological data show that joint prenatal exposure to stress and Pb can result in an increased risk of adverse neurodevelopmental outcomes not observed in animals exposed to either stressor independently [11]. It has been hypothesized that the neurodevelopmental effects observed in toxicological studies examining joint Pb and stress exposure may be a result of shared toxicity targets, including the hypothalamic-pituitary-adrenal (HPA) axis and the dopamine and glutamatergic systems [11,12]. We identified no studies that examined joint exposure to Pb, MeHg, and stress in reproductive-aged women or any other populations.

Although blood levels of Pb and MeHg (half-lives of approximately 1 and 2 months, respectively) are reliable surrogates of recent exposures [13,14], quantifying chronic stress exposure is more challenging. Stress is a consequence of perceiving an “exposure” (e.g., violence, poverty) as more than one can handle (*i.e*., “stressful”) [15]. Humans respond to stressful exposures via measurable physiological changes mediated by the HPA axis [16]. Chronic stress can result in physiological dysregulation of the stress response including both primary mediators (*i.e*., cortisol and the catecholamines) and secondary outcome measures (e.g., blood pressure, C-reactive protein), which can be quantitatively estimated via allostatic load (AL) [16,17]. AL has been defined as the number of physiological measures (*i.e.,* primary mediators and secondary outcomes) across multiple systems (e.g.*,* cardiovascular, metabolic, immune), responding outside of the normal range [16].

We examine race/ethnicity as a predictor of joint neurodevelopmental toxicant (NDT) exposure to Pb and MeHg in a nationally-representative sample of nonpregnant, reproductive-aged, women. Additionally, AL is used as an indicator of chronic stress exposure and examined as a potential effect measure modifier of the association between joint elevated NDT exposure and race/ethnicity. 

## 2. Methods

### 2.1. The National Health and Nutrition Examination Surveys (NHANES) Data Set

All biomarker (chemical and nonchemical stressor exposures), sociodemographic, lifestyle, and nutrient status data used in this analysis were extracted from NHANES. NHANES is a continuous survey that includes a physical examination (*i.e*., medical, dental, and physiological measurements), laboratory tests and an interview component collecting demographic, socioeconomic, dietary, and health-related data representative of the Unites States (U.S.) population. A stratified, multistage probability sample of the civilian, non-institutionalized U.S. population is used to select participants [18]. The 2003−2004 NHANES included 10,102 individuals including 5,152 women. For our analysis of female NHANES participants, the following exclusion criteria were applied: (1) not of reproductive age ([*n* = 3,331 [reproductive age is defined here as 15 to 44 years]), (2) did not complete both the questionnaire and physical examinations (*n* = 64); (3) were pregnant (*n* = 347), (4) self-identified their race/ethnicity as “other Hispanic” or “other race” (*n* = 104), or (5) were missing biomarkers for blood Pb or blood MeHg (*n* = 56). The final data set included 1,250 participants.

### 2.2. Chemical NDT Stressor Exposure

Whole blood Hg (total and inorganic) and Pb concentrations were determined using inductively coupled plasma-mass spectrometry [19]. Because inorganic Hg was below the detection limit (0.3 µg/L) in 75% of participants and total and inorganic Hg had different detection limits, we assumed that total Hg was entirely MeHg. The detection limits were 0.2 µg/dL for Pb and 0.14 µg/L for total Hg.

To evaluate joint Pb and MeHg exposure, we assumed dose-addition and calculated a hazard index (HI) for the combined Pb and MeHg dose by summing individual hazard quotients (HQs) [20]. Dose-addition assumes that both Pb and MeHg affect the same target, here the nervous system. HQs were calculated by comparing the blood Pb and blood MeHg biomarker concentrations with an NDT-specific health reference value (HRV). The following HRVs were used: HRV_Pb_ = 1.76 µg/dL, and HRV_MeHg_ = 5.8 µg/L. The HRV_MeHg_ was based on the maternal blood concentration associated with the U.S. Environmental Protection Agency’s (EPA’s) MeHg reference dose (RfD) [21,22]. The HRV_Pb_ was based on a prospective cohort study reporting no intelligence quotient decrements at 3 years of age in Polish males with cord blood Pb values ≤ 1.21 µg/dL, compared with males having cord blood Pb values > 1.21 µg/dL [23]. Using an average maternal-to-cord blood Pb ratio of 1.45 [24], we calculated the maternal blood HRV_Pb_ (1.76 µg/dL) equivalent to the cord blood HRV_Pb_ (1.21 µg/dL).

For the *i^th^* participant and the *j^th^* NDT, the NDT concentration was compared with the NDT-specific HRV to generate an HQ; these were summed across the two NDTs (Pb and MeHg) to evaluate joint NDT exposure for the *i^th^* participant (Equation 1):

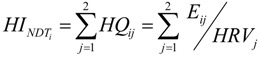
(1)
where *HI_NDTi_* is the hazard index from both Pb and MeHg for the *i^th^* participant; *HQ_ij_* is the hazard quotient for the *j^th^* NDT and the *i^th^* participant; *E_ij_* is the blood biomarker concentration for the *j^th^* NDT and the *i^th^* participant; and *HRV_j_* is the blood health reference value for the *j^th^* NDT.

All hazard values (*i.e*., HQ_Pb_, HQ_MeHg_, HI_NDT_) were dichotomized with values >1 used as an indicator of elevated exposure (e.g., an HI_NDT_ > 1 indicates elevated joint exposure to Pb and MeHg).

Unlike the RfD-based HRV_MeHg_, our literature-derived HRV_Pb_ does not contain an uncertainty factor to account for intra-species variability. Therefore, we conducted a sensitivity analysis using lower and higher HRV_Pb_ values based on California EPA’s blood Pb benchmark in children of 1.0 µg/dL [25] and the Centers for Disease Control and Prevention’s (CDC’s) reference level of 5.0 µg/dL [26].

### 2.3. AL Biomarkers

The following ten AL biomarkers were chosen based on multi-system representation, availability, and previous research [27]: heart rate, mean systolic and diastolic blood pressure, homocysteine, body mass index (BMI), high-density lipoprotein (HDL) and total cholesterol, glycohemoglobin, C-reactive protein, and albumin (see online Supplementary Information for additional details). To evaluate potential chronic stress exposure, we computed an AL-clinical_i_ score for the *i^th^* participant by summing across 10 biomarker indicator variables (BIV_ik_) where the *k^th^* biomarker was assigned a “1” if its clinical criterion was exceeded or a “0” if not [28] (Equation 2, see online Supplementary Information, Tables S1 and S2):

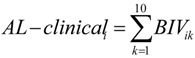
(2)

AL-clinical scores were used as an indicator of chronic stress exposure with scores of 0; 1 or 2; and ≥3, assumed to correspond to low, intermediate, and high chronic stress exposure, respectively. These cut-points were based on the distribution of AL-clinical scores and represent 28, 54, and 20%, of women respectively. Participants missing AL biomarker data (*n* = 79) were excluded from analyses unless existing AL biomarker data led to an AL-clinical score of ≥3 (*n* = 11).

Other methods for calculating AL scores, including using empirical cut-points for classifying high risk and summation of *z*-scores, reportedly yield similar results [27,29,30]. We conducted a sensitivity analysis, calculating AL scores using empirically-determined high risk cut-points (AL-empirical) based on the distribution of each AL biomarker with high risk corresponding to the third quartile―high risk for HDL cholesterol and albumin correspond to the first quartile as lower values of these biomarkers indicate increased risk (see online Supplementary Information, Tables S1–S3).

### 2.4. Data Analysis

Statistical summaries were generated for all individual biomarkers and summary indices (*i.e*., AL, HQ_Pb_, HQ_MeHg_, HI_NDT_) (see Tables S1 and S2). Logistic regression was used to evaluate the association between elevated joint NDT exposure (HI_NDT_ ≥ 1) and race/ethnicity. Confounding was evaluated using univariate and bivariate analyses (data not shown). Confounders were included in the multivariate model if they changed the association between elevated joint NDT exposure and race/ethnicity by ≥10% (see online Supplementary Information for a complete list of confounders and associated details). Chronic stress exposure was examined as an effect measure modifier via an interaction term in the final model (race/ethnicity × AL) and stratifying the multivariate model by AL-clinical groups. NHANES two-year sampling weights were used to produce unbiased, national estimates. Sampling weights do not account for differential birth rates, which could impact analyses. To evaluate the potential impact of differential birth rates on the associated between elevated joint NDT exposure and race/ethnicity, we performed an additional sensitivity analysis accounting for race/ethnicity- and age-specific birth rates based on methods from Axelrad and Cohen [31]; NHANES two-year sampling weights were multiplied by race/ethnicity- and age-specific birth rates and used in place of the original NHANES two-year sampling weights that were used in the main analysis.

All statistical analyses were conducted using SAS^®^ software v9.3 (SAS Institute, Cary. NC, USA). Following NHANES analytic guidelines [18], we used procedures that accurately incorporated the stratification and multistage sampling: Proc SurveyMeans^®^, Proc SurveyLogistic^®^, and Proc SurveyFreq^®^ survey procedures. Statistical significance was set at an alpha level of 0.05 and examined by the use of *p-*values and 95% Wald confidence intervals (95% CIs).

## 3. Results

### 3.1. Pb and MeHg Concentrations and Joint NDT Exposure

Study participants were more likely to be Caucasian (weighted percentage = 74%), born in the U.S. (90%), greater than 28 years of age (58%), college educated (58%), nonsmokers (72%), and have a normal iron status (74%) (see Table 1). Overall median (± interquartile range) Pb and MeHg blood concentrations were 0.9 ± 0.6 µg/dL and 0.8 ± 1.0 µg/L, respectively. The median HQ_Pb _(0.5 ± 0.3) was greater than the median HQ_MeHg_ (0.1 ± 0.1). Caucasians had a lower median HQ_Pb_ compared to both African Americans and Mexican Americans (*p* < 0.05), but no race/ethnicity differences were detected for HQ_MeHg_. The median HI_NDT_ was 0.7 ± 0.5. As with HQ_Pb_, Caucasians had a lower median HI_NDT_ compared to both African Americans and Mexican Americans (*p* < 0.01; see online Supplementary Information, Tables S1 and S2).

### 3.2. Elevated Joint NDT Exposure and Chronic Stress

Overall, 11% of participants had elevated blood Pb levels (HQ_Pb_ > 1), with Mexican Americans (22%) having a higher proportion compared to Caucasians (9%) (*p* < 0.05; see Table 1). Only 2% of all participants had elevated blood MeHg levels; no differences were detected by race/ethnicity. One-quarter of all study participants had elevated joint NDT exposure levels and 20% were classified as having a high AL, with African Americans having higher proportions compared to Caucasians for both elevated NDT exposure and high AL (*p* < 0.05).

Adjusting for country of birth, age, education, smoking status, and iron status had a mixed effect on the association between elevated joint NDT exposure and race/ethnicity. The odds ratio (OR) increased nearly 30% for African Americans after accounting for relevant confounders (unadjusted OR [95% CI] = 1.7 [1.0, 2.6]; adjusted OR [95% CI] = 2.2 [1.4, 3.3]) but decreased by 43% for Mexican Americans (unadjusted OR [95% CI] = 2.0 [1.3, 3.0]; adjusted OR [95% CI] = 1.4 [0.7, 2.6]) (see Table 2). In addition to differences detected by race/ethnicity in the multivariate model, participants were more likely to have elevated joint NDT exposure if they were (1) foreign born (adjusted OR [95% CI] = 3.3 [1.6, 6.8]); (2) older, including 20–28 years of age (1.8 [1.0, 3.2]) and 29–44 years of age (3.5 [2.4, 5.1]); or (3) smokers (3.0 [1.7, 5.0]). An inverse association was detected between elevated joint NDT exposures and iron status, with participants having an abnormal iron status less likely to have elevated joint NDT exposure (adjusted OR [95% CI] = 0.6 [0.5, 0.8]).

### 3.3. Effect Measure Modification by AL

**Figure 1:**
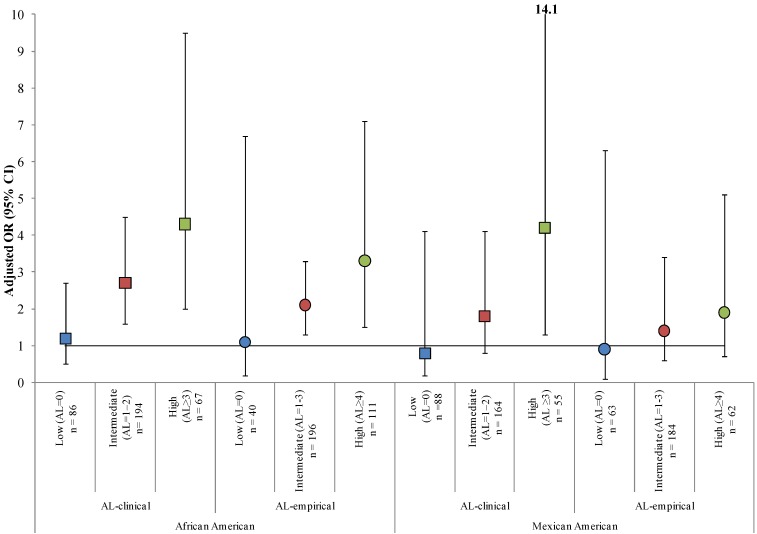
Adjusted ^a^ odds ratios (ORs) and 95% Wald confidence intervals (CIs) for the association between elevated joint neurodevelopmental toxicant (NDT) exposure ^b^ and race/ethnicity stratified by allostatic load^c^ among nonpregnant, reproductive-aged (15−44 years) women from NHANES 2003−2004.

Notes: ^a^ Adjusted for age, country of birth, head of household highest education, smoking status, and iron status. ^b^ Elevated NDT exposure was defined as having a hazard index (HI) for joint lead (Pb) and methyl mercury (MeHg) greater than one (HI_NDT_ > 1). The HI was calculated by summing individual hazard quotients (HQs) for Pb and MeHg (Equation 1). HQs were calculated by dividing blood concentrations of Pb and MeHg by 1.76 µg/dL and 5.8 µg/L, respectively. ^c^ Allostatic load (AL), a measure of physiological dysregulation, was used as an indicator of chronic stress exposure and was estimated based on the categorical classification of AL scores and calculated by summing the number of 10 biomarkers above clinical (AL-clinical) or empirical (AL-empirical) high risk criteria (Equation 2). Squares indicate adjusted ORs for AL-clinical. Circles indicate adjusted ORs for AL-empirical. Regardless of AL method: blue indicates low AL (low chronic stress exposure), red indicates intermediate AL (intermediate chronic stress exposure), and green indicates high AL (high chronic stress exposure).

Effect measure modification of the association between elevated joint NDT exposure and race/ethnicity was detected by AL. The overall interaction term race/ethnicity × AL was not statistically significant (*p* = 0.1) but the interaction between African Americans and high AL was statistically significant (*p* = 0.02). Although no racial/ethnic differences were observed among participants with low ALs, the largest ORs were noted among participants with high ALs (adjusted OR [95% CI] = 4.3 [2.0, 9.5] and 4.2 [1.3, 14.1] for African Americans and Mexican Americans, respectively) (see Table 2, Figure 1). Although an increasing trend across AL groups was not detected for age, ORs increased with increasing age for all AL groups with the largest ORs observed among participants with high ALs (adjusted ORs [95% CI] = 19.1 [1.8, 201.6] and 33.3 [3.6, 305.5] for ages 20–28 years and 29–44 years, respectively). An inverse association between country of birth and elevated joint NDT exposure was observed across AL groups among foreign-born participants with the largest ORs observed among foreign-born participants with low ALs (adjusted OR [95% CI] = 10.3 [2.5, 43.2]).

### 3.4. Sensitivity Analyses

The main analysis assumed equal birth rates across all race/ethnicity and age categories; therefore, we performed a sensitivity analysis accounting for different birth rate trends across race/ethnicity and age. Using NHANES two-year sampling weights adjusted for race/ethnicity—and age-specific birth rates resulted in similar trends as noted in the main analysis but lower ORs for the association between elevated joint NDT exposure and race/ethnicity particularly among Mexican Americans with high AL-clinical scores (see online Supplementary Information, Table S4).

We also examined differences in ORs using AL-empirical scores and lower and higher HRV_Pb_s (1.0 µg/dL and 5.0 µg/dL, respectively). We found comparable relationships for African Americans using AL-empirical scores (see online Supplementary Information, Table S3). Larger ORs were noted for the high AL group using a HRV_Pb_ of 5.0 µg/dL, but the results were less stable. Mexican Americans with a high AL tended to have larger ORs, compared to other AL groups, regardless of the AL approach (AL-clinical or AL-empirical) or HRV used (1.0 µg/dL and 5.0 µg/dL), except when AL-empirical scores and the higher HRV_Pb_ were used in combination (see online Supplementary Information, Table S3).

**Table 1:** Characteristics of nonpregnant, reproductive-aged (15–44 years) women from NHANES 2003−2004 by indicators of elevated neurodevelopmental toxicant (NDT) exposure ^a^ and allostatic load ^b^.

	All Women *n* (Weighted %)	Indicators of Elevated NDT Exposure ^a^			Allostatic Load ^b^		
		HQ_Pb_ > 1 *n* (Weighted %)	HQ_MeHg_ > 1 *n* (Weighted %)	HI_NDT_ > 1 *n* (Weighted %)	Low *n* (Weighted %)	Intermediate *n* (Weighted %)	High *n* (Weighted %)
Total Population	1,250	159 (11)	19 (2)	324 (25)	330 (28)	632 (54)	210 (20)
**Race/Ethnicity**							
Caucasian	551 (74)	46 (9)	10 (2)	119 (22)	156 (28) *	274 (54)	88 (19)
African American	373 (15)	52 (14)	6 (2)	104 (32) *	86 (18)	194 (55)	67 (26) *
Mexican American	326 (11)	61 (22) *	3 (1)	101 (36) *	88 (21)	164 (56)	55 (23)
**Country of Birth**							
United States	1,049 (90)	92 (8)	15 (2) *	225 (23)	278 (25)	533 (54)	175 (20)
Foreign	201 (10)	67 (33) *	4 (2)	99 (48) *	52 (26)	99 (52)	35 (22)
**Age (years)**							
15−19	520 (16)	43 (5)	4 (1)	79 (11)	191 (43)	254 (49)	40 (8)
20−28	252 (26)	29 (9)	3 (1)	59 (20) *	66 (30) *	136 (58) *	31 (12) *
29−44	478 (58)	87 (14) *	12 (2)	184 (33) *	73 (19) *	242 (54)	139 (28) *
**Highest Education ^c^**							
Less than high school graduate	327 (15)	70 (22) *	4 (1)	114 (39)	68 (18) *	170 (53)	69 (29) *
High school graduate	302 (27)	37 (11)	3 (1)	67 (21)	74 (20) *	152 (53)	55 (27) *
Some college	383 (35)	30 (7)	3 (1)	75 (19)	107 (28) *	197 (56)	54 (17)
College graduate or above	194 (23)	17 (8)	9 (5) *	57 (31) *	67 (33)	90 (54)	26 (13)
**Smoking Status (serum cotinine)**							
Nonsmoker (≤10 ng/mL)	943 (72)	100 (8)	14 (2)	214 (22)	270 (27)	485 (54)	147 (19)
Smoker (>10 ng/mL)	282 (28)	56 (18) *	5 (2)	105 (37) *	60 (21) *	147 (55)	62 (25) *
**Iron Status Indicator ^d^**							
Normal	870 (74)	105 (11)	17 (2)	235 (28)	247 (27) *	449 (55)	122 (18) *
Abnormal	379 (26)	54 (11)	2 (0)	89 (21)	83 (19)	183 (52)	87 (28)

Notes: ^a^ Hazard quotients (HQs) were calculated by dividing blood concentrations of lead (Pb) and methyl mercury (MeHg) by 1.76 µg/dL and 5.8 µg/L, respectively. The hazard index (HI) was calculated by summing individual hazard quotients for Pb and MeHg (Equation 1). ^b^ Allostatic load, a measure of physiological dysregulation, was used as an indicator of chronic stress exposure and was estimated based on the categorical classification of AL-clinical scores (0 = Low; 1−2 = Intermediate; ≥3 = High) and calculated by summing the number of 10 biomarkers above clinical high risk criteria (Equation 2). ^c^ If the participant was <18 years of age, head of household status was used. ^d^ Iron status was determined abnormal if any two of the following conditions were met: (1) serum ferritin <15 ng/mL, (2) transferrin saturation <16%, (3) red blood cell distribution width >15%, or (4) erythrocyte protoporphyrin >50 µg/dL red blood cells. * Denotes statistically significant within group differences between the indicated percentage and the lowest (or highest) percentage (*p* < 0.05) (e.g., Mexican Americans had a statistically significant higher percentage of women with an HQ_Pb_ > 1 relative to Caucasians).

**Table 2:** Odds Ratios (ORs) and 95% Wald Confidence Intervals (CIs) for the association between elevated joint neurodevelopmental toxicant exposure ^a^ and race/ethnicity among nonpregnant, reproductive-aged (15−44 years) women from NHANES 2003−2004.

	OR (95% CI)				
	Univariate	Multivariate	Multivariate by Allostatic Load ^b^		
			Low	Intermediate	High
		*n* = 1,181	*n* = 316	*n* = 609	*n* = 203
**Race/Ethnicity**	*n* = 1,250				
Caucasian	1.0	1.0	1.0	1.0	1.0
African American	1.7 (1.0, 2.6)	2.2 (1.4, 3.3)	1.2 (0.5, 2.7)	2.7 (1.6, 4.5)	4.3 (2.0, 9.5)
Mexican American	2.0 (1.3, 3.0)	1.4 (0.7, 2.6)	0.8 (0.2, 4.1)	1.9 (0.9, 4.0)	4.2 (1.3, 14.1)
**Country of Birth**	*n* = 1,250				
United States	1.0	1.0	1.0	1.0	1.0
Foreign	3.1 (1.8, 5.4)	3.3 (1.6, 6.8)	10.3 (2.5, 43.2)	2.2 (1.2, 4.3)	1.8 (0.4, 8.1)
**Age (years)**	*n* = 1,250				
15−19	1.0	1.0	1.0	1.0	1.0
20−28	2.1 (1.4, 3.2)	1.8 (1.0, 3.2)	2.5 (0.9, 7.4)	1.6 (0.6, 4.2)	19.1 (1.8, 201.6)
29−44	4.1 (3.1, 5.5)	3.5 (2.4, 5.1)	6.3 (2.2, 17.7)	3.9 (1.8, 8.4)	33.3 (3.6, 305.5)
**Highest Education ^c^**	*n*= 1,250				
Less than high school graduate	1.4 (0.8, 2.5)	0.8 (0.5, 1.5)	1.9 (0.5, 7.6)	0.7 (0.3, 1.7)	0.6 (0.1, 2.9)
High school graduate	0.6 (0.3, 1.0)	0.4 (0.2, 0.6)	0.6 (0.2, 1.8)	0.4 (0.2, 0.6)	0.5 (0.1, 4.3)
Some college	0.5 (0.3, 0.8)	0.8 (0.5, 1.5)	0.8 (0.4, 1.7)	0.2 (0.1, 0.4)	0.7 (0.2, 2.8)
College graduate or above	1.0	1.0	1.0	1.0	1.0
**Smoking Status (serum cotinine)**	*n* = 1,225				
Nonsmoker (≤10 ng/mL)	1.0	1.0	1.0	1.0	1.0
Smoker (>10 ng/mL)	2.0 (1.4, 3.0)	3.0 (1.7, 5.0)	2.0 (0.8, 4.6)	3.8 (2.0, 7.4)	3.4 (1.1, 10.6)
**Iron Status Indicator^d^**	*n* = 1,250				
Normal	1.0	1.0	1.0	1.0	1.0
Abnormal	0.7 (0.5, 1.0)	0.6 (0.5, 0.8)	1.1 (0.5, 2.4)	0.5 (0.3, 0.7)	0.7 (0.3, 1.4)

Notes: ^a^ Elevated NDT exposure was defined as having a hazard index (HI) for joint lead (Pb) and methyl mercury (MeHg) greater than one (HI_NDT_ > 1). The HI was calculated by summing individual hazard quotients (HQs) for Pb and MeHg (Equation 1). HQs were calculated by dividing blood concentrations of Pb and MeHg by 1.76 µg/dL and 5.8 µg/L, respectively. ^b^ Allostatic load (AL), a measure of physiological dysregulation, was used as an indicator of chronic stress exposure and was estimated based on the categorical classification of AL-clinical scores (0 = Low; 1−2 = Intermediate; >2 = High) and calculated by summing the number of 10 biomarkers above clinical high risk criteria (Equation 2). ^c^ If the participant was <18 years of age, status of the head of household was used. ^d^Iron status was determined to be abnormal if any two of the following conditions were met: (1) serum ferritin <15 ng/mL, (2) transferrin saturation <16%, (3) red blood cell distribution width >15%, or (4) erythrocyte protoporphyrin >50 µg/dL red blood cells.

## 4. Discussion

Our analysis suggests that 25% of nonpregnant, reproductive-aged women in the 2003−2004 NHANES had blood levels of combined Pb and MeHg that may be of concern for adverse neurodevelopment in offspring (see Table 1). We identified race/ethnicity as one of the strongest individual-level predictors of elevated joint NDT exposure to Pb and MeHg. While numerous studies have estimated exposure distributions of Pb and MeHg independently [32,33], consideration of the joint exposure distribution of these NDTs is rare [5,6]. Our study also found that AL, an indicator of chronic stress, modified the association between race/ethnicity and elevated joint NDT exposure (see Table 2; Figure 1). We found no differences across racial/ethnic groups for the likelihood of having elevated joint NDT exposure among participants with low chronic stress. Whether chronic stress increases vulnerability to Pb- and/or MeHg-mediated neurodevelopmental toxicity is unclear. Our analysis, however, provides evidence of joint exposure to chronic stress and multiple NDTs and suggests that racial/ethnic disparities exist for both chemical and nonchemical exposures independent of other risk factors including age and education.

The HI is a screening approach that examines the joint hazard associated with multiple chemicals under an assumption of dose-addition and is useful for the preliminary prioritization of stressors [20]. There is limited information available regarding the joint neurotoxicity of Pb and MeHg; therefore, there is uncertainty associated with the assumption of dose-addition but additivity is consistent with CDC recommendations for this combination of NDTs [34]. Although the current analysis only examined a chemical mixture of two metals with a similar endpoint, this approach could be extended to include additional chemical stressors. It is currently unclear whether nonchemical stressors, such as measures of physiological dysregulation, can be directly incorporated into the HI. In general, effect measure modification can be used to examine vulnerable populations and identify potential interactions. We examined effect measure modification by potential chronic stress exposure on the main effect using stratification and tests for interactions.

The HRVs used in the HI calculation for the current analysis are similar to Biomonitoring Equivalents in that biomarker concentrations are compared with risk assessment-based values to facilitate their interpretation in a public health risk context [35]. The HRV_MeHg_ was based on the U.S. EPA RfD. RfDs are “estimates of daily exposure for the human population… likely to be without an appreciable risk of deleterious effects over a lifetime”; such values have been thoroughly peer reviewed [36]. Because no scientific consensus exists on a “safe value” of blood Pb, we chose a value identified in the literature that is unlikely to be associated with adverse neurodevelopment. The HRV_Pb_ used in the current analysis has not been as thoroughly vetted as the HRV_MeHg_ and does not include an uncertainty factor accounting for intra-person variability. We conducted sensitivity analyses using higher and lower HRV_Pb_s to highlight some of the uncertainty associated with our HRV_Pb_ and found similar results across all HRV_Pb_s.

The HI is health conservative, identifying populations estimated to have an HQ > 1 for any single chemical, as well as < 1 for all chemicals individually, yet have an HI > 1 when HQs are combined across stressors. For example, half of the 25% of women identified as having elevated joint NDT exposure, based on an HI_NDT_ > 1, had HQs < 1 for Pb or MeHg, and less than 1% of these women (*n* = 3) had HQs > 1 for both Pb and MeHg independently. Additionally, the HI approach accounts for the relative toxicities of stressors, potentially allowing for prioritization of stressors and identification of vulnerable populations. This is exemplified in our analysis: Pb accounted for more than 70% of the total HI_NDT_ and African Americans were more likely than Caucasians to experience elevated joint NDT exposure. Thompson and Boekelheide [5] reported that, although statistically significant differences by race/ethnicity were not observed for the odds of greater than median exposure to Pb, MeHg, or PCBs, non-Caucasians had larger adjusted ORs compared to Caucasians, and Hispanics had smaller adjusted ORs compared to other non-Caucasians. Our results underscore the importance of monitoring chemical and nonchemical stressor exposures.

Exposure disparities across and within racial/ethnic groups may be associated with differential vulnerability. Vulnerability can imply that an individual or population differs from the “general” population because of increased exposure, a different dose-response curve, or a differential ability to resist or recover from stressor exposures [37]. For example, we found that, although 36% of all Mexican American women had elevated joint NDT exposure (see Table 1), there were statistically significant differences among Mexican Americans by country of birth (U.S. born = 23% and foreign-born = 51%; unadjusted OR [95% CI] = 3.6 [2.2, 6.1], data not shown). Nelson, *et al.* [38] also reported differences in environmental chemical concentrations between foreign-born and U.S. born Mexican Americans. Although Crimmins, *et al.* [39] found that U.S. born Mexican Americans had higher ALs compared to both Caucasians and foreign-born Mexican Americans, we did not detect statistically significant differences among Mexican Americans by country of birth for the likelihood of having high chronic stress. Although country of birth was a statistically significant variable in our multivariate model and a decrease in adjusted ORs was noted with increasing AL score (see Table 2), we did not have a large enough sample size to further investigate the potential interaction between race/ethnicity and country of birth and any modifying effect by potential chronic stress exposure. A strength of this study was the large number of variables available for examination as possible confounders of the main effect (see online Supplementary Information for a complete list); therefore, the potential for residual confounding was decreased but perhaps not entirely eliminated as some variables of interest may be mismeasured and data on other possible confounders were not available (e.g., occupation, genetic susceptibility, *etc*.). Results from our analysis and previous studies [5,38,39] reinforce the need to examine chemical and nonchemical stressor exposures across and within sociodemographics in future epidemiological studies and cumulative risk assessments (CRAs) as these factors may contribute to differential vulnerability.

Sociodemographic risk factors may also influence birth rates [40]. Using sampling weights adjusted for race/ethnicity- and age-specific birth rates, we observed comparable results to the main analysis, but most ORs were attenuated (see online Supplementary Information, Table S4). The smaller adjusted ORs were likely due to the combination of different birth rates, NDT exposures and AL scores across racial/ethnic groups. For example, although the percentage of women with elevated joint NDT exposure and high chronic stress increased with age across all racial/ethnic groups, birth rates were higher among younger non-Caucasian and older Caucasian women. While the use of sampling weights adjusted for race/ethnicity- and age-specific birth rates minimally affected the logistic regression results, the estimated number of women with elevated joint NDT exposure was lower (only about 8% of the estimate based on unadjusted birth rates, data not shown). This sensitivity analysis supports the main results that African American women with high chronic stress had higher odds of elevated joint NDT exposure. This analysis also highlights the importance of accounting for differential outcome rates (e.g., birth rates) when estimating associations and/or the number of individuals potentially at increased risk.

Exposure measurement is a primary challenge in epidemiological studies and CRAs. Cross-sectional data, such as NHANES, have limitations that can limit their utility in broader applications. For example, the potential for reverse causation due to uncertain temporality (*i.e*., ensuring that a putative causal agent preceded the exposure) is often a concern. This was not an issue in our analysis as we were assessing whether race/ethnicity, which is an immutable factor that is constant over time, was a predictor of elevated joint NDT exposure. There is, however, some uncertainty due to the assumption that NDT exposures detected from one-time measures of Pb and MeHg in non-pregnant reproductive age women are representative of potential future maternal and *in utero* exposures. Both Pb and MeHg have relatively short half-lives in blood (approximately 1 and 2 months, respectively) [13,14]; therefore, concentrations in the current analysis reflect recent exposures. Additionally, chemical stressor exposures likely differ during pregnancy. For example, blood Pb may increase during pregnancy due to increased bone turnover [41]. In contrast, levels of many environmental chemicals are reportedly lower among pregnant women [42] potentially due to physiological changes (e.g., increased blood volume) and lifestyle and behavioral modifications. The use of one-time biomarker samples can result in measurement error, which may limit our ability to fully elucidate independent associations and effect measure modification that may be evident in this population.

In addition to the limitations associated with one-time measurements, AL scores, used to indicate chronic stress exposure, were limited by the lack of availability of the primary stress mediators (*i.e.*, cortisol, catecholamines), although these biomarkers also have limitations [43]. Measures of physiological dysregulation, such as AL, are not ideal indicators of chronic stress exposure because non-chronic stress factors such as genetics and lifestyle choices could also result in physiological dysregulation and, therefore, a higher AL [16]. Regardless of the cause(s) associated with an increased AL, physiological dysregulation may be associated with increased vulnerability to adverse effects associated with exposure to chemical stressors [44]. We excluded pregnant women from our analysis because of uncertainty whether AL could serve as a surrogate of *in utero* chronic stress exposure due to the physiological changes that occur throughout pregnancy [45,46]. However, it is reasonable to assume that nonpregnant women of reproductive age identified as having elevated levels of neurodevelopmental stressors could also have elevated levels during pregnancy, and this may be an indication of potential concern for their offspring.

Psychosocial stressors are difficult to measure, as evidenced by various reported approaches for assessing chronic stress; such as exposure to violence [47], the University of California, Los Angeles Life Stress Interview [48], the Perceived Stress Scale [49], depression [50], and AL [27]. No NHANES interview questions among reproductive-aged women pertain to chronic stress; therefore, we used AL as an indicator of chronic stress exposure. Although quantitative measures of physiological dysregulation, such as AL, may decrease reporting error and increase reproducibility and comparability across studies, having subjective measures of chronic stress, such as those listed above, would be useful for cross-validation. There are, however, no standard clinical high risk criteria, and empirical high risk cut-points vary depending on the underlying population characteristics, making it difficult to compare results across studies and to make generalizations across populations. In our analysis, empirical cut-points were influenced by Caucasians, the predominant population in NHANES. Caucasians had lower values for nearly all AL biomarkers compared to African Americans but similar values to Mexican Americans (see online Supplementary Information, Table S2). Therefore, we believe that AL-clinical, as used in our main analysis, was better suited for evaluating racial/ethnic differences.

Additional limitations have been associated with the operationalization of AL [27,51]. Traditional AL approaches dichotomize AL biomarkers, but there is likely a continuum of risk that may not lend itself well to dichotomous classification. An associated limitation is not accounting for the bi-directionality of physiological dysregulation (*i.e.*, assuming that potential concern exists only above a defined cut-point). Levels outside the normal physiological range (higher or lower) may be associated with dysregulation and chronic stress (e.g., low BMI) [52]. Future research should examine whether AL is a useful measure of chronic stress across the lifespan, especially in reproductive-aged women before, during, and after pregnancy, as well as account for the bi-directionality and continuum of harm associated with individual AL biomarkers.

## 5. Conclusions

Chemical and nonchemical stressors are rarely examined jointly in human health studies or risk assessments. Our results suggest that chronic stress modified the association between race/ethnicity and elevated joint NDT exposure. These results support the use of existing methods (e.g., HI and AL) and data sources (e.g., NHANES) to examine cumulative chemical and nonchemical stressor exposures, potentially elucidating causal associations and explaining equivocal results reported across epidemiological studies for some environmental chemicals. Including chronic stress in the evaluation of joint NDT exposure allowed for the identification of potentially vulnerable populations, which may require further evaluation.

## Supplementary Files

Supplementary File 1 Supplementary Information (PDF, 98 KB)
