# Supplementary material for: Joint Exposure to Chemical and Nonchemical Neurodevelopmental Stressors in U.S. Women of Reproductive Age in NHANES
Source: Int J Environ Res Public Health. 2014 Apr 22;11(4):4384–401. doi: 10.3390/ijerph110404384 (PMC4024991; doi:10.3390/ijerph110404384)
Supplement: Supplementary File 1 — Supplementary Information (PDF, 98 KB) [file ijerph-11-04384-s001.pdf]

Supplementary Information

Joint Exposure to Chemical and Nonchemical Neurodevelopmental Stressors in U.S. Women of Reproductive Age in NHANES

1. Methods

1.1. Allostatic Load (AL) Biomarkers

Details regarding measurement procedures are described within the following National Health and Nutrition Examination Survey (NHANES) manuals: *Anthropometry Procedures Manual* [1] for height and weight used in the calculation of body mass index (BMI), *Cardiovascular Fitness Procedures Manual* [2] for heart rate and blood pressures, and *Laboratory Procedure Manuals* for homocysteine in plasma [3], high density lipoprotein (HDL) and total cholesterol [4], glycohemoglobin in whole blood [5], albumin in refrigerated serum [6], and C-reactive protein in serum [7].

Clinical high risk cut-points are listed in Table S1. Cardiovascular markers included heart rate (pulse), systolic and diastolic blood pressure, and homocysteine. Heart rate was measured in beats per minute and the normal clinical range for all adults is 60–100 beats per minute according to the National Institute of Health (NIH) [8]; therefore the clinical high risk cut-point was >100 beats per minute. Mean blood pressure was calculated as follows: if only one measurement was available, this was the average; if two measurements were available, the first measurement was discarded and the second was used as the average; if more than two measurements were available, the first measurement was discarded and remaining measurements were averaged. We used only mean blood pressure readings to classify blood pressure as high or low risk regardless of the use of blood pressure medication. Clinical high risk values for blood pressure were >130 mmHg systolic and >85 mmHg diastolic according to HeartHealthyWomen.org [9], which is a joint project of the Cardiovascular Research Foundation, the Office on Women's Health of the Department of Health and Human Services, and WomenHeart: the National Coalition for Women with Heart Disease. Fasting homocysteine was measured in plasma and is considered to have a normal range of 5–15 μmol/L [9]; therefore, the clinical high risk cut-point was >15 μmol/L. Metabolic biomarkers included BMI, HDL and total cholesterol, and glycohemoglobin. High risk cholesterol levels were specific for females with HDL cholesterol levels < 50 mg/dL and total cholesterol levels ≥ 200 mg/dL considered high risk [9]. BMI ≥ 25 [9] and glycohemoglobin ≥ 6.5% [10] were considered high risk. Inflammatory biomarkers included C-reactive protein and albumin with levels ≥ 1 mg/dL [9] and ≤ 3.4 [8] considered high risk, respectively.

1.2. Sensitivity Analysis: Allostatic Load Score

For the empirical based AL (AL-Empirical), data from all women, including those excluded from the final analytical sample due to missing data for any of the 10 AL biomarkers, were used to estimate weighted percentiles for each biomarker (see Supplementary Information, Table S1). Biomarkers were

considered "high risk" if above the 75th percentile and the biomarker indicator variable (BIV) received a value of "1", with the exception of HDL cholesterol and albumin for which higher values are deemed more health-protective. Therefore, these two biomarkers were considered "high risk" above the 25th percentile. BIVs for the empirical method were summed to calculate the AL-Empirical score. AL-Empirical score were categorized using the same approach that was used to categorize AL-Clinical scores (Equation 2, Tables S1 and S2).

1.3. Potential Confounders and Variable Classification

We used univariate (each variable was included in the model as the only independent variable) and bivariate (race/ethnicity and one additional variable were included in the model as independent variables) analyses to examine the following variables as potential confounders of the association between an indicator of elevated neurodevelopmental toxicant (NDT) exposure and race/ethnicity: country of birth (United States *vs.* foreign; status of the head of household was used if participant was <18 years of age), age (years, continuous and categorical: 15–19, 20–28, 29–44), marital status (married or living with partner; never married; widowed, divorced, or separated; status of the head of household was used if participant was <18 years of age and never married), annual household income (<$19,999; ≥$19,999−<$45,000, ≥$45,000−<$75,000; ≥$75,000), annual family income (<$14,999; ≥$14,999−<$35,000, ≥$35,000−<$75,000; ≥$75,000), poverty-to-income ratio (continuous and categorical: <1; 1−<2; ≥2−<3; ≥3), highest educational attainment (less than high school or high school graduate; some college/associates or technical degree; college graduate or above; status of head of household was used if participant was <18 years of age), home owned or rented, smoking status (serum cotinine: ≤10 ng/mL = nonsmoker; >10 ng/mL = smoker), physical activity (combination of any activity daily and vigorous activity monthly: both, either, none), type of home (attached, detached), year home was built (<1940–1959, 1960–1977, 1978–1989, 1990–present), how many years lived in home (<1, 1–2, 3–5, 6–10, >10), iron status indicator (normal *vs.* abnormal). Abnormal iron status was defined as meeting any two of the following conditions: (1) serum ferritin < 15 ng/mL, (2) transferrin saturation < 16%, (3) red blood cell distribution width > 15%, (4) erythrocyte protoporphyrin > 50 µg/dL red blood cells [11–13].

Table S1. Descriptive statistics from individual allostatic load (AL) biomarkers, multisystem AL scores [a] for multiple approaches, and screening-level indicators of joint neurodevelopmental toxicant (NDT) exposure [b] to lead (Pb) and methyl mercury (MeHg), using multiple health reference values (HRVs) for Pb, among nonpregnant, reproductive-aged (15–44 years) survey participants from NHANES 2003–2004.

		**All Women**				
				**Quartiles**		
	**HRV [c]**	***n***	**Range**	**25th**	**50th**	**75th**
**Cardiovascular Markers**						
Heart Rate (beats/min) **	>100	1,250	44–128	68	76	84
Mean Systolic BP (mm Hg) *, #	≥130	1,250	78–178	102	110	117
Mean Diastolic BP (mm Hg) #	≥85	1,250	24–116	62	67	74
Homocysteine (μmol/L) **, #	>15	1,243	2.9–23.9	5.9	6.8	8.0
**Metabolic Markers**						
Body Mass Index (kg/m^2) *, **, #	≥25	1,250	15–63	22	26	31
HDL-Cholesterol (mg/dL) **, #	<50	1,224	23–123	46	56	66
Total Cholesterol (mg/dL) *	≥200	1,224	94–650	160	182	211
Glycohemoglobin (%) *, **	≥6.5	1,246	4.2–13.2	5.0	5.1	5.3
**Immune Markers**						
C-reactive protein (mg/dL) **	≥1.0	1,233	0.01–7.9	0.06	0.18	0.49
Albumin (g/dL) *, #	≤3.4	1,218	3.2–5.2	4.0	4.2	4.4
**NDTs**						
Pb (μg/dL) *, **	1.8	1,250	0.2–23.9	0.7	0.9	1.3
MeHg (μg/L)	5.8	1,250	0.1–30.7	0.4	0.8	1.4
**Multisystem AL [a]**						
AL-Clinical *, **		1,162	0–6	0	1	2
AL-Empirical *, #		1,410	0–8	1	2	3

Table S1. *Cont.*

	All Women				
			Quartiles		
**HRV [c]**	***n***	**Range**	**25th**	**50th**	**75th**
**Screening-Level Indicators of NDT Exposure [b]**					
HQ_{PB} *,**	1,250	0.1–13.7	0.4	0.5	0.7
HQ_{PB} (lower HRV_{Pb}) *,**	1,250	0.2–23.9	0.7	0.9	1.3
HQ_{Pb} (higher HRV_{Pb}) *,**	1,250	0.04–4.8	0.1	0.2	0.3
HQ_{MeHg}	1,250	0.02–5.3	0.07	0.1	0.2
HI_{NDT} *,**	1,250	0.1–13.7	0.5	0.7	1.0
HI_{NDT} (lower HRV_{Pb}) *,**	1,250	0.2–24.0	0.8	1.1	1.6
HI_{NDT} (higher HRV_{Pb}) *	1,250	0.06–5.5	0.3	0.4	0.5

Notes: HQ = hazard quotient; HI = hazard index. [a] AL scores were calculated by summing the number of 10 biomarkers that were above clinical or empirical high risk cut-points for AL-Clinical and AL-Empirical, respectively. [b] HQs were calculated by dividing biomarker concentrations by chemical-specific HRVs, HI_{NDTs} were calculated by summing the individual HQs for Pb and MeHg. [c] AL biomarkers = Clinical high risk cut-points, MeHg = EPA's blood equivalent of the RfD, Pb = maternal blood equivalent (1.76 µg/dL) of the cord blood literature derived value [14] 1.0 µg/dL and 5.0 µg/dL were used as the lower and higher HRV_{Pb}, respectively. * Denotes statistically significant mean differences ($p < 0.05$) between African Americans and Caucasians. ** Denotes statistically significant differences ($p < 0.05$) between Mexican Americans and Caucasians. #Denotes statistically significant differences ($p < 0.05$) between African Americans and Mexican Americans.

Table S2. Descriptive statistics from individual allostatic load (AL) biomarkers, multisystem AL scores [a] for multiple approaches, and screening-level indicators of joint neurodevelopmental toxicant (NDT) exposure [b] to lead (Pb) and methyl mercury (MeHg), using multiple health reference values (HRVs) for Pb, among nonpregnant, reproductive-aged (15–44 years) survey participants from NHANES 2003–2004 by race/ethnicity.

	Caucasians					African Americans					Mexican Americans				
			Quartiles					Quartiles					Quartiles		
	n	**Range**	**25th**	**50th**	**75th**	***n***	**Range**	**25th**	**50th**	**75th**	***n***	**Range**	**25th**	**50th**	**75th**
**Cardiovascular Markers**															
Heart Rate (beats/min) **	557	44–114	70	76	84	389	48–128	68	76	84	329	50–106	66	72	82
Mean Systolic BP (mm Hg) **, #	555	78–158	102	109	116	381	82–173	105	114	123	324	83–178	102	109	115
Mean Diastolic BP (mm Hg) #	555	31–103	62	67	74	381	36–105	62	68	77	324	24–116	60	66	73
Homocysteine (µmol/L) **, #	548	3.4–23.9	6.0	6.9	8.2	371	2.9–14.9	5.7	6.8	8.0	324	3.2–15.5	5.4	6.2	7.3
**Metabolic Markers**															
Body Mass Index (kg/m^2) *, **, #	566	15–54	21	25	30	387	15–63	24	30	35	335	16–54	23	27	33
HDL-Cholesterol (mg/dL) **, #	538	25–123	47	57	67	367	27–118	46	55	65	322	23–107	44	52	61
Total Cholesterol (mg/dL) *	538	99–650	161	183	213	364	102–305	157	181	202	322	94–306	156	180	206
Glycohemoglobin (%) *, **	549	4.5–9.2	4.9	5.1	5.2	371	4.2–9.7	5.1	5.3	5.5	326	4.5–13.2	5.1	5.2	5.4
**Immune Markers**															
C-reactive protein (mg/dL) **	543	0.01–4.7	0.06	0.2	0.4	368	0.01–6.5	0.07	0.3	0.7	322	0.01–7.9	0.09	0.3	0.7
Albumin (g/dL) *, #	535	3.3–5.2	4.0	4.3	4.5	362	3.2–4.9	3.8	4.0	4.2	321	3.3–5.0	4.0	4.2	4.5
**NDTs**															
Pb (µg/dL) *, **	551	0.2–5.0	0.6	0.9	1.2	373	0.2–9.5	0.8	1.1	1.4	326	0.2–23.9	0.7	1.2	1.7
MeHg (µg/L)	551	0.1–15.6	0.4	0.7	1.4	373	0.1–8.2	0.6	0.9	1.7	326	0.1–30.7	0.4	0.6	1.1
**Multisystem AL [a]**															
AL-Clinical *, **	572	0–5	0	1	2	396	0–6	1	2	2	338	0–5	1	2	2
AL-Empirical *, #	572	0–8	1	2	3	396	0–8	2	3	4	338	0–7	1	2	4

Table S2. *Cont.*

	Caucasians					African Americans					Mexican Americans				
			Quartiles					Quartiles					Quartiles		
	n	**Range**	**25th**	**50th**	**75th**	***n***	**Range**	**25th**	**50th**	**75th**	***n***	**Range**	**25th**	**50th**	**75th**
**Screening-Level Indicators of NDT Exposure** [b]															
HQ_{PB} [*,**]	551	0.1–2.9	0.3	0.5	0.7	373	0.1–5.4	0.5	0.6	0.8	326	0.1–13.7	0.4	0.7	1.0
HQ_{PB} (lower HRV_{Pb}) [*,**]	551	0.2–5.0	0.6	0.9	1.2	373	0.2–9.5	0.8	1.1	1.4	326	0.2–23.9	0.7	1.2	1.7
HQ_{Pb} (higher HRV_{Pb}) [*,**]	551	0.04–1.0	0.1	0.2	0.2	373	0.04–1.9	0.2	0.2	0.3	326	0.04–4.8	0.1	0.2	0.3
HQ_{MeHg}	551	0.02–2.7	0.07	0.1	0.2	373	0.02–1.4	0.1	0.2	0.3	326	0.02–5.3	0.07	0.1	0.2
HI_{NDT} [*,**]	551	0.1–3.4	0.5	0.7	1.0	373	0.2–5.9	0.6	0.8	1.1	326	0.2–13.7	0.5	0.8	1.2
HI_{NDT} (lower HRV_{Pb}) [*,**]	551	0.2–5.1	0.8	1.1	1.5	373	0.3–9.6	1.0	1.3	1.7	326	0.3–24.0	0.9	1.3	1.9
HI_{NDT} (higher HRV_{Pb}) [*]	551	0.06–2.9	0.2	0.3	0.5	373	0.08–2.8	0.3	0.4	0.6	326	0.08–5.5	0.2	0.4	0.5

Notes: HQ = hazard quotient; HI = hazard index. [a] AL scores were calculated by summing the number of 10 biomarkers that were above clinical or empirical high risk cut-points for AL-Clinical and AL-Empirical, respectively. [b] HQs were calculated by dividing biomarker concentrations by chemical-specific HRVs, HI_{NDTs} were calculated by summing the individual HQs for Pb and MeHg. [c] AL biomarkers = Clinical high risk cut-points, MeHg = EPA's blood equivalent of the RfD, Pb = maternal blood equivalent (1.76 µg/dL) of the cord blood literature derived value [14] 1.0 µg/dL and 5.0 µg/dL were used as the lower and higher HRV_{Pb}, respectively.[*] Denotes statistically significant mean differences ($p < 0.05$) between African Americans and Caucasians. [**] Denotes statistically significant differences ($p < 0.05$) between Mexican Americans and Caucasians. [#] Denotes statistically significant differences ($p < 0.05$) between African Americans and Mexican Americans.

Table S3. Unadjusted and adjusted [a] odds ratios (with 95% Wald confidence intervals) for the association between elevated neurodevelopmental toxicant exposure [b] and race/ethnicity among nonpregnant, reproductive-aged (15–44 years) survey participants from NHANES 2003–2004 stratified by sensitivity analyses using different health reference values (HRVs) for lead (Pb) [b] and different approaches for calculating allostatic load (AL) scores [c].

		Caucasians	African Americans			Mexican Americans		
			**HRV Pb (1.76)**	**HRV Pb (1.0)**	**HRV Pb (5.0)**	**HRV Pb (1.76)**	**HRV Pb (1.0)**	**HRV Pb (5.0)**
**Univariate**		REF	1.8 (1.1, 2.8)	2.1 (1.4, 3.2)	1.3 (0.6, 3.3)	1.9 (1.2, 2.9)	1.6 (1.1, 2.5)	1.4 (0.5, 3.5)
**Multivariate** [b]		REF	2.2 (1.4, 3.3)	2.6 (1.6, 4.4)	1.5 (0.6, 3.6)	1.4 (0.7, 2.6)	1.3 (0.9, 1.9)	0.7 (0.2, 1.9)
**AL-Clinical**	**Low**	REF	1.2 (0.5, 2.7)	1.0 (0.3, 2.6)	1.6 (0.4, 6.6)	0.8 (0.2, 4.1)	1.1 (0.5, 2.4)	0.6 (0.1, 2.4)
	**Intermediate**	REF	2.7 (1.6, 4.5)	4.2 (1.9, 9.4)	1.3 (0.4, 4.5)	1.8 (0.8, 4.1)	1.4 (0.9, 2.4)	2.3 (0.5, 9.5)
	**High**	REF	4.3 (2.0, 9.5)	3.4 (1.5, 7.6)	8.3 (0.9, 72.8)	4.2 (1.3, 14.1)	2.2 (1.0, 4.6)	2.9 (0.7, 11.4)
**AL-Empirical**	**Low**	REF	1.1 (0.2, 6.7)	1.5 (0.4, 5.9)	1.1 (0.1, 9.9)	0.9 (0.1, 6.3)	1.5 (0.6, 3.9)	5.3 (0.2, 157.6)
	**Intermediate**	REF	2.1 (1.3, 3.3)	2.3 (1.3, 3.9)	0.6 (0.2, 1.5)	1.4 (0.6, 3.4)	1.1 (0.6, 2.0)	0.7 (0.2, 2.8)
	**High**	REF	3.3 (1.5, 7.1)	6.7 (2.4, 19.0)	39.0 (3.1, 497.4)	1.9 (0.7, 5.1)	2.7 (1.5, 4.9)	0.2 (0.008, 4.3)

Notes: Results highlighted in aqua are the main analysis results. [a] Adjusted for age, country of birth, education, smoking status, iron status. [b] Elevated neurodevelopmental toxicant exposure was defined as having a hazard index for joint lead and methyl mercury greater than one (HINDT > 1). The hazard index was calculated by summing individual hazard quotients for lead and methyl mercury (Equation 1). Hazard quotients were calculated by dividing blood concentrations of lead and methyl mercury by 1.76 µg/dL and 5.8 µg/L, respectively. The lower HRV for Pb was 1.0 µg/dL [15] and the higher HRV for Pb was 5.0 µg/dL [16]. [c] AL scores were calculated by summing the number of 10 biomarkers that were above clinical or empirical high risk cut-points for AL-Clinical and AL-Empirical, respectively. Both AL approaches were categorized based on the distribution of AL scores (AL-Clinical: low = 0, intermediate = 1–2, high = 3–6; AL-Empirical: low = 0, intermediate = 1–3, high = 4–8).

Table S4. Adjusted odds ratios (ORs) and 95% confidence intervals (CIs) for the association between elevated neurodevelopmental toxicant exposure [a] and race/ethnicity stratified by allostatic load [b] among nonpregnant, reproductive-aged (15–44 years) survey participants from NHANES 2003–2004 using 2-year sample weights adjusted for race/ethnicity-age-specific birth rates.

	**OR (95% CI)**			
	**Multivariate**	**Multivariate by Allostatic Load [b]**		
	n = 1,181	**Low**	**Intermediate**	**High**
		n = 316	n = 609	n = 203
**Race/Ethnicity**				
Caucasian	REF	REF	REF	REF
African American	1.7 (1.0, 3.0)	1.3 (0.5, 3.3)	1.7 (0.8, 3.4)	3.2 (1.2, 8.8)
Mexican American	1.1 (0.5, 2.4)	1.3 (0.2, 10.0)	1.0 (0.3, 3.0)	1.4 (0.2, 9.6)
**Country of Birth**				
United States	REF	REF	REF	REF
Foreign	3.4 (1.7, 6.8)	8.0 (1.9, 34.0)	2.7 (1.4, 5.1)	3.0 (0.4, 22.7)
**Age (years)**				
15–19	REF	REF	REF	REF
20–28	1.9 (1.1, 3.0)	2.5 (1.0, 6.1)	1.5 (0.7, 3.2)	16.0 (1.7, 152.8)
29–44	3.6 (2.7, 4.7)	6.6 (2.5, 17.1)	3.6 (2.0, 6.5)	23.0 (3.0, 177.0)
**Highest Education [c]**				
Less than high school graduate/GED	1.1 (0.6, 1.9)	2.0 (0.6, 7.1)	1.0 (0.4, 2.3)	0.7 (0.1, 4.7)
High School graduate/GED	0.7 (0.4, 1.2)	1.4 (0.3, 7.1)	0.5 (0.3, 0.9)	0.5 (0.05, 4.7)
Some college/AA degree	0.4 (0.2, 0.8)	1.3 (0.4, 4.0)	0.2 (0.1, 0.5)	0.3 (0.1, 1.9)
College graduate or above	REF	REF	REF	REF
**Smoking Status (serum cotinine)**				
Nonsmoker (≤10 ng/mL)	REF	REF	REF	REF
Smoker (>10 ng/mL)	2.0 (1.3, 3.2)	2.4 (0.8, 6.7)	1.9 (0.9, 3.8)	2.1 (0.6, 7.9)
**Iron Deficiency Indicator [d]**				
Normal	REF	REF	REF	REF
Abnormal	0.6 (0.4, 1.0)	0.5 (0.2, 1.2)	0.7 (0.5, 1.1)	0.7 (0.3, 1.4)

Notes: [a] Elevated neurodevelopmental toxicant exposure was defined as having a hazard index for joint lead and methyl mercury greater than one ($HI_{NDT} > 1$). The hazard index was calculated by summing individual hazard quotients for lead and methyl mercury. Hazard quotients were calculated by dividing blood concentrations of lead and methyl mercury by 1.76 µg/dL and 5.8 µg/L, respectively. [b] Allostatic load was used as a surrogate of chronic stress and was estimated based on the categorical classification of allostatic load scores (0 = Low; 1–2 = Intermediate; >2 = High) calculated by summing the number of 10 biomarkers above clinical high risk criteria (Equation 2). [c] Head of household status was used if participant was < 18 years of age. [d] Iron status was determined to abnormal if any two of the following conditions were met: (1) serum ferritin < 15 ng/mL, (2) transferrin saturation < 16%, (3) red blood cell distribution width > 15%, (4) erythrocyte protoporphyrin > 50 µg/dL red blood cells.

References

1. Centers for Desease Control and Prevention (CDC). National Health and Nutrition Examination Survey: Anthropometry Procedures Manual; CDC: Atlanta, GA, USA, 2004.
2. Centers for Desease Control and Prevention (CDC). *National Health and Nutrition Examination Survey: Cardiovascular Fitness Procedures Manual*; CDC National Center for Health Statistics: Atlanta, GA, USA, 2004.
3. Centers for Desease Control and Prevention (CDC). *Laboratory Procedure Manual: Total Homocysteine (tHcy) in Plasma. NHANES 2003–2004*; CDC National Center for Health Statistics: Atlanta, GA, USA, 2005.
4. Centers for Desease Control and Prevention (CDC). *Laboratory Procedure Manual: Total Cholesterol, HDL-Cholesterol, Triglycerides, and LDL-Cholesterol. NHANES 2003–2004*; CDC National Center for Health Statistics: Atlanta, GA, USA, 2013.
5. Centers for Desease Control and Prevention (CDC). *Glycohemoglobin in Blood. NHANES 2003–2004*; CDC National for Health Statistics: Atlanta, GA, USA, 2005.
6. Centers for Desease Control and Prevention (CDC). *Laboratory Procedure Manual: Albumin in Refrigerated Serum. NHANES 2003–2004*; CDC National Center for Health Statistics: Atlanta, GA, USA, 2005.
7. Centers for Desease Control and Prevention (CDC). *Laboratory Procedure Manual: C-Reactive Protein in Serum. NHANES 2003–2004*; CDC National Center for Health Statistics: Atlanta, GA, USA, 2000.
8. MedlinePLus. *NLM Pulse*; U.S. National Library of Medicine: Bethesda, MD, USA, 2013.
9. National Institutes of Health (NIH). *The Healthy Heart Handbook for Women*; No. 07–2720; NIH: Bethesda, MD, USA, 2007.
10. American Diabetes Association. Executive summary: Standards of medical care in diabetes—2013. *Diabetes Care* **2013**, *36*, S4–S10.
11. World Health Organization (WHO). Iron Deficiency Anaemia: Assessment, Prevention, and Control., A Guide for Programme Managers; WHO: Geneva, Switzerland, 2001.
12. Centers for Disease Control and Prevention (CDC). Iron-Status Indications. National Report on Biochemical Indicators of Diet and Nutritionin the U.S. Populaiton 1999–2002; CDC: Atlanta, GA, USA, 2008.
13. McClung, J.; Marchitelli, L.; Friedl, K.; Young, A. Prevalence of iron deficiency and iron deficiency anemia among three populations of female military personnel in the US Army. *J. Am. Coll. Nutr.* **2006**, *25*, 64–69.
14. Jedrychowski, W.; Perera, F.; Jankowski, J.; Mrozek-Budzyn, D.; Mroz, E.; Flak, E.; Edwards, S.; Skarupa, A.; Lisowska-Miszczyk, I. Gender specific differences in neurodevelopmental effects of prenatal exposure to very low-lead levels: The prospective cohort study in three-year olds. *Early Hum. Dev.* **2009**, *85*, 503–510.
15. Carlisle, J.C.; Dowling, K.C.; Siegel, D.M.; Alexeeff, G.V. A blood lead benchmark for assessing risks from childhood lead exposure. *J. Environ. Sci Health A* **2009**, *44*, 1200–1208.

16. Centers for Disease Control and Prevention (CDC). *What Do Parents Need to Know to Protect. Their Children?* CDC: Atlanta, GA, USA, 2012.

© 2014 by the authors; licensee MDPI, Basel, Switzerland. This article is an open access article distributed under the terms and conditions of the Creative Commons Attribution license (http://creativecommons.org/licenses/by/3.0/).
